# Functional Brain Networks Develop from a “Local to Distributed” Organization

**DOI:** 10.1371/journal.pcbi.1000381

**Published:** 2009-05-01

**Authors:** Damien A. Fair, Alexander L. Cohen, Jonathan D. Power, Nico U. F. Dosenbach, Jessica A. Church, Francis M. Miezin, Bradley L. Schlaggar, Steven E. Petersen

**Affiliations:** 1 Behavioral Neuroscience Department, Oregon Health and Science University, Portland, Oregon, United States of America; 2 Department of Neurology, Washington University School of Medicine, St. Louis, Missouri, United States of America; 3 Department of Radiology, Washington University School of Medicine, St. Louis, Missouri, United States of America; 4 McDonnell Center for Higher Brain Functions, Washington University School of Medicine, St. Louis, Missouri, United States of America; Indiana University, United States of America

## Abstract

The mature human brain is organized into a collection of specialized functional networks that flexibly interact to support various cognitive functions. Studies of development often attempt to identify the organizing principles that guide the maturation of these functional networks. In this report, we combine resting state functional connectivity MRI (rs-fcMRI), graph analysis, community detection, and spring-embedding visualization techniques to analyze four separate networks defined in earlier studies. As we have previously reported, we find, across development, a trend toward ‘segregation’ (a general decrease in correlation strength) between regions close in anatomical space and ‘integration’ (an increased correlation strength) between selected regions distant in space. The generalization of these earlier trends across multiple networks suggests that this is a general developmental principle for changes in functional connectivity that would extend to large-scale graph theoretic analyses of large-scale brain networks. Communities in children are predominantly arranged by anatomical proximity, while communities in adults predominantly reflect functional relationships, as defined from adult fMRI studies. In sum, over development, the organization of multiple functional networks shifts from a local anatomical emphasis in children to a more “distributed” architecture in young adults. We argue that this “local to distributed” developmental characterization has important implications for understanding the development of neural systems underlying cognition. Further, graph metrics (e.g., clustering coefficients and average path lengths) are similar in child and adult graphs, with both showing “small-world”-like properties, while community detection by modularity optimization reveals stable communities within the graphs that are clearly different between young children and young adults. These observations suggest that early school age children and adults both have relatively efficient systems that may solve similar information processing problems in divergent ways.

## Author Summary

The first two decades of life represent a period of extraordinary developmental change in sensory, motor, and cognitive abilities. One of the ultimate goals of developmental cognitive neuroscience is to link the complex behavioral milestones that occur throughout this time period with the equally intricate functional and structural changes of the underlying neural substrate. Achieving this goal would not only give us a deeper understanding of normal development but also a richer insight into the nature of developmental disorders. In this report, we use computational analyses, in combination with a recently developed MRI technique that measures spontaneous brain activity, to help us to understand the principles that guide the maturation of the human brain. We find that brain regions in children communicate with other regions more locally but that over age communication becomes more distributed. Interestingly, the efficiency of communication in children (measured as a ‘small world’ network) is comparable to that of the adult. We argue that these findings have important implications for understanding both the maturation and the function of neural systems in typical and atypical development.

## Introduction

The mature human brain is both structurally and functionally specialized, such that discrete areas of the cerebral cortex perform distinct types of information processing. These areas are organized into functional networks that flexibly interact to support various cognitive functions. Studies of development often attempt to identify the organizing principles that guide the maturation of these functional networks. [1]–[6].

A major portion of the work investigating the nature of functional human brain development is based on results from functional magnetic resonance imaging (fMRI) studies. By examining the differences in the fMRI activation profile of a particular brain region between children, adolescents, and adults, the developmental trajectory of that region's involvement in a cognitive task can be outlined [3], [5], [7]–[10]. These experiments have been crucial to our current understanding of typical and atypical brain development.

In addition to fMRI activation studies, the relatively new and increasingly utilized method of resting state functional connectivity MRI (rs-fcMRI) allows for a complementary examination of the functional relationships between regions across development. Resting state fcMRI is based on the discovery that spontaneous low-frequency (<∼0.1 Hz) blood oxygen level dependent (BOLD) signal fluctuations in sometimes distant, but functionally-related grey matter regions, show strong correlations at rest [11]. These low frequency BOLD fluctuations appear to relate to spontaneous neural activity [11]–[13]. In effect, rs-fcMRI evaluates regional interactions that occur when a subject is not performing an explicit task (i.e., subjects are “at rest”) [11], [12], [14]–[23]. To date, rs-fcMRI has been used in several domains to examine systems-level organization of motor [11], memory [24],[25], attention [26], and task control systems [21],[22],[27].

In addition, because rs-fcMRI does not require active engagement in a behavioral task, it unburdens experimental design, subject compliance, and training demands. Thus, rs-fcMRI is becoming a frequently used tool for examining changes in network structure in disease [28]–[31], in aging [24],[29], and across development [22], [32]–[35].

### Resting-state fcMRI identifies separable brain networks in adults

**Figure 1:**
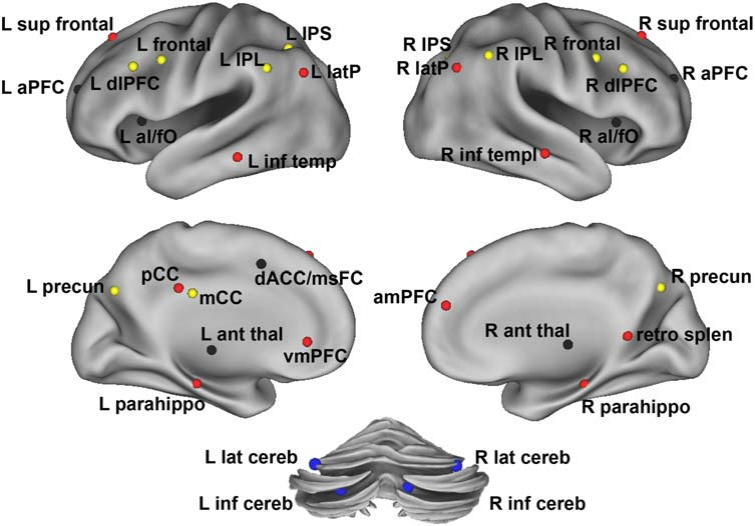
Anatomical location of regions shown in Table 1. Regions are colored by network membership (red – default mode network; black – cingulo-opercular network; yellow – fronto-parietal network; blue – cerebellar network) and shown on an inflated cortical surface represention.

In previous work regarding task-level control in adults, we applied rs-fcMRI to a set of regions derived from an fMRI meta-analysis that included studies of control-demanding tasks. This analysis revealed that brain regions exhibiting different combinations of control signals across many tasks are grouped into distinct “fronto-parietal” and “cingulo-opercular” functional networks [21],[36] (see Table 1 and Figure 1). Based on functional activation profiles of these regions characterized in the previous fMRI study, the fronto-parietal network appears to act on a shorter timescale, initiating and adjusting top-down control. In contrast, the cingulo-opercular network operates on a longer timescale providing “set-initiation” and stable “set-maintenance” for the duration of task blocks [37].

**Table 1:** Regions, coordinates, and properties.

Regions of Interest (ROI)	ROI Abbreviations	Coordinates			Functional Network	Network Color
		*x*	*y*	*z*		
dorsolateral prefrontal cortex	dlPFC	−43	22	34	Fronto_Parietal	Yellow
dorsolateral prefrontal cortex	dlPFC	43	22	34	Fronto_Parietal	Yellow
Frontal	frontal	−41	3	36	Fronto_Parietal	Yellow
Frontal	frontal	41	3	36	Fronto_Parietal	Yellow
mid cingulate cortex	mCC	0	−29	30	Fronto_Parietal	Yellow
inferior parietal lobule	IPL	−51	−51	36	Fronto_Parietal	Yellow
inferior parietal lobule	IPL	51	−47	42	Fronto_Parietal	Yellow
intraparietal sulcus	IPS	−31	−59	42	Fronto_Parietal	Yellow
intraparietal sulcus	IPS	30	−61	39	Fronto_Parietal	Yellow
Precuneus	Precun	−9	−72	37	Fronto_Parietal	Yellow
Precuneus	Precun	10	−69	39	Fronto_Parietal	Yellow
anterior Prefrontal Cortex	aPFC	−28	51	15	Cingulo_Opercular	Black
anterior Prefrontal Cortex	aPFC	27	50	23	Cingulo_Opercular	Black
anterior insula/frontal operculum	aI/fO	−35	14	5	Cingulo_Opercular	Black
anterior insula/frontal operculum	aI/fO	36	16	4	Cingulo_Opercular	Black
dorsal anterior cingulate/medial superior frontal cortex	dACC/msFC	−1	10	46	Cingulo_Opercular	Black
superior frontal cortex	ant thal	−12	−15	7	Cingulo_Opercular	Black
anterior thalamus	ant thal	10	−15	8	Cingulo_Opercular	Black
anterior thalamus	amPFC	1	54	21	Default	Red
ventromedial prefrontal cortex	vmPFC	−3	39	−2	Default	Red
superior frontal cortex	sup frontal	−14	38	52	Default	Red
superior frontal cortex	sup frontal	17	37	52	Default	Red
inferior temporal	inf templ	−61	−33	−15	Default	Red
inferior temporal	inf templ	65	−17	−15	Default	Red
parahippocampal	parahippo	−22	−26	−16	Default	Red
parahippocampal	parahippo	25	−26	−14	Default	Red
posterior cingulate cortex	pCC	−2	−36	37	Default	Red
lateral parietal	latP	−47	−67	36	Default	Red
lateral parietal	latP	53	−67	36	Default	Red
retro splenial	retro splen	3	−51	8	Default	Red
lateral cerebellum	lat cereb	−32	−66	−29	Cerebellar	Blue
lateral cerebellum	lat cereb	31	−61	−29	Cerebellar	Blue
inferior cerebellum	inf cereb	−19	−78	−33	Cerebellar	Blue
inferior cerebellum	inf cereb	18	−80	−33	Cerebellar	Blue

Along with these two task control networks [21],[36], a set of cerebellar regions showing error-related activity across tasks [36] formed a separate cerebellar network (Figure 1). In adults, the cerebellar network is functionally connected with both the fronto-parietal and cingulo-opercular networks [21],[22]. These functional connections may represent the pathways involved in task level control that provide feedback information to both control networks [22],[36].

Another functional network, and one of the most prominent sets of regions to be examined with rs-fcMRI, is the “default mode network”. The default mode network (frequently described as being composed of the bilateral posterior cingulate/precuneus, inferior parietal cortex, and ventromedial prefrontal cortex) was first characterized by a consistent decrease in activity during goal-directed tasks compared to baseline [38],[39]. Resting-state fcMRI analyses have repeatedly shown that these regions, along with associated medial temporal regions, are correlated at rest in adults [15],[16],[32],[40]. While the distinct function of the default mode network is often linked to internally directed mental activity [39], this notion continues to be debated [25], [32], [41]–[44].

### Spontaneous correlated activity within brain networks develops over age

In two prior developmental studies, we used rs-fcMRI to examine the development of the task control and cerebellar functional networks [22] and, separately, the default mode network [32]. The first study, addressing functional connectivity changes within and between the two task control networks and the cerebellar network [22], showed that the structure of these networks differed between children and adults in several ways (see [22]). In general, many of the specific changes showed trends of decreases in short-range functional connections (i.e., correlations between regions close in space) and increases in long-range functional connections (i.e., correlations between regions more distant in space). We suggested that these global developmental processes support the maturation of a dual-control system and its functional connections with the cerebellar network [22]. These results have now been replicated in a developmental resting connectivity study targeting sub-regions of the anterior cingulate [34].

The development of the default mode network was independently examined in a separate analysis [32]. In children, the default mode network was only sparsely functionally connected. Many regions were relatively isolated with few or no functional connections to other default mode regions. Over age, correlations within the default mode network increased and by adulthood it had matured into a fully integrated system. Interestingly, as opposed to the task-control and cerebellar networks, very few short-range functional connections involving the default mode network regions existed in children. Hence the numerous strong short-range functional connections that decreased with age when investigating the dual control networks were not seen within the default network. In fact, some connections such as the functional connection between the ventromedial prefrontal cortex (vmPFC; −3, 39, −2) and anterior medial prefrontal cortex (amPFC; 1, 54, 21) regions, which are fairly close in space (i.e., short-range at ∼2.7 cm), had a substantial increase in correlation strength over development [32].

The observation that different analyses suggested different developmental features suggests a need for a more nuanced and integrated characterization of the development of functional networks. The goal of this manuscript is to employ several different network analysis tools to provide such a characterization. Visualization techniques such as spring embedding, and quantitative measures, including ‘small world’ metrics and community detection algorithms, will be applied to these networks in an attempt to identify principles for the changes observed across development.

Because of the overlapping and sometimes inconsistent use of terminology between neuroscience and the computational sciences, we will briefly define two terms for the purposes of this paper. The term “networks” will be used in the typical cognitive neuroscience formulation: a group of functionally related brain regions (as described above). The overall collection of regions (encompassing all four “networks”) will be referred to as the “graph.”

## Results

### Spring-embedded visualization in combination with functional connectivity suggests that regions are linked more locally in childhood and are more distributed in adulthood

Graph theory analyses were applied to 210 subjects, aged 7–31, to investigate the emergence of temporal correlations in spontaneous BOLD activity between regions of the default mode, cerebellar, and two task-control networks. For this initial analysis, average age-group matrices were created using a sliding boxcar grouping of subjects in age-order (i.e., group1: subjects 1–60, group2: subjects 2–61, group3: subjects 3–62, etc.). This generated a series of groups with average ages ranging from 8.48 years to 25.58 years. Each of the groups' average correlation matrices was converted into a graph, with correlations between regions greater than or equal to 0.1 considered as functionally connected.

**Figure 2:**
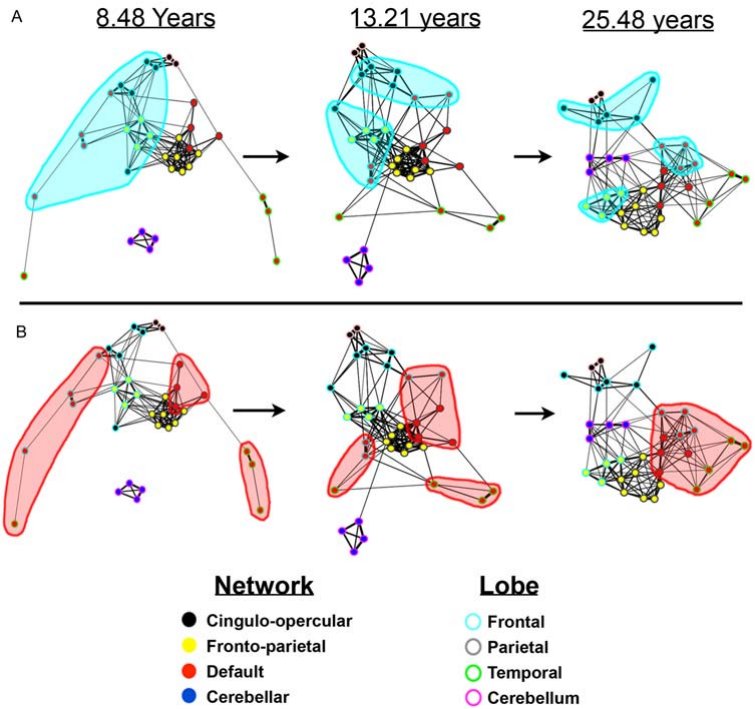
Over age the graph architecture matures from a “local” organization to a “distributed” organization. In this figure we show the dynamic development and interaction of positive correlations between the two task control networks, the default network, and cerebellar network using spring embedding. The figure highlights the segregation of local, anatomically clustered regions and the integration of functional networks over development. A and B represent individual screen shots (at average ages 8.48, 13.21, and 25.48 years) of dynamic movies (Video S1) of the transition in the network architecture from child to adult ages. Nodes are color coded by their adult network profile (core of the nodes) and also by their anatomical location (node outlines). Black – cingulo-opercular network; Yellow – fronto-parietal network; Red – default network; Blue – cerebellar network; Light blue – frontal cortex; Grey – parietal cortex; Green - temporal cortex, Pink – cerebellum, Light pink – thalamus. Connections with r≥0.1 were considered connected. (A) In children regions are largely organized by their anatomical location, but over age anatomically clustered regions segregate. The cluster of frontal regions (highlighted in light blue) best demonstrates this *segregation*. (B) In children the more distributed adult functional networks are in many ways disconnected. Over development the functional networks integrate. The isolated regions of the default mode network in childhood (highlighted in light red) that coalesce into a highly correlated network best illustrate this *integration*. Over age node organization shifts from the “local” arrangement in children to the “distributed” organization commonly observed in adults.

In a first analysis, we used a visualization algorithm commonly used in graph theoretic analyses known as spring embedding that aids in the qualitative interpretation of graphs (Figure 2 and Video S1) [45]. In spring embedding, the positions of the nodes (i.e., regions) in a graph are based solely on the strength and pattern of functional connections instead of their anatomical locations. In this procedure, each functional connection between a pair of nodes is treated as a spring with a spring constant related to the strength of the specific correlation. The entire system of pair-wise regional functional connections is then iteratively allowed to relax to the lowest global energetic state, i.e., groups of nodes that are strongly interconnected will be placed close together even if anatomically distant.

By creating spring embedded graphs for each of the sliding boxcar groups in age-order, a movie representation can be made that shows the development of the network relationships (from average age 8.48 to 25.48 years) (Video S1). The panels in Figure 2 provide snapshots from child, adolescent, and adult average ages in this movie. In both Figure 2 and Video S1, each node is color-coded in two ways: the outer border represents the general anatomical location (i.e., cerebral lobe) of the node; the inner core color represents the coding by “function” as defined by a large number of fMRI studies.

One of the primary observations from the movie relates to this anatomical-functional distinction. In children, regions appear to be largely arranged by anatomical proximity. This arrangement can be seen in Figure 2 and Video S1 where, in children, regions can be readily grouped by cerebral lobe (outline colors of spheres in Figure 2 and Video S1). Over age, as functional connections mature, the node arrangements change such that anatomically close regions are now largely distributed across the graph layout, in a pattern more aligned with the mature networks' functional properties (core colors of spheres in Figure 2) [21], [36]–[39]. Thus, across development, local clusters of regions “segregate” from one another and “integrate” into more distributed adult functional relationships with more distant regions.

A group of regions in the frontal cortex provides a particularly salient example of segregation. Frontal cortex contains regions that, in adults, are members of each of the task-control networks (e.g., dlPFC, frontal, dACC/msFC) and the default network (e.g., vmPFC, amPFC). As can be seen in Figure 2A (and Video S1), extensive correlations exist between most of these frontal regions in childhood (see blue cloud Figure 2A). Over the developmental window afforded by the current dataset, some of these strong “frontal-frontal” correlations begin to weaken. With increasing age, regions in the frontal cluster segregate into 3 separate functional networks.

Accompanying this segregation is strong integration within the functional networks. The default mode network provides the clearest example. As illustrated in Figure 2B (and in Video S1), correlations between regions of the default mode network are weak (or absent) in children (red cloud, Figure 2B). Just as functional connections between the set of frontal regions are related to their anatomical proximity in children, the regions of the default mode network are each functionally connected to anatomical neighbors, and not to other members of the anatomically dispersed default mode network. Over age, however, the functional connections between default mode network regions mature and the network integrates into a highly correlated system in adults (Figure 2B and Video S1) (also see [32]). We note that these results were not specific to the 60-subject boxcar, and persist with smaller subject boxcars as well (see Video S2).

### Quantitative modularity analysis confirms the qualitative observations

The qualitative observations noted above can be quantified using community structure detection tools. Using such an approach is particularly important because of the bias inherent in relying on qualitative methods for deciding whether groups of regions that appear to be clustered are indeed clustered, and because of the *a priori* definitions of each network. As stated by Newman:

“A good division of a graph into communities is not merely one in which there are few edges between communities; it is one in which there are fewer than expected edges between communities. If the number of edges between two groups is only what one would expect on the basis of random chance, then few thoughtful observers would claim this constitutes evidence of meaningful community structure. On the other hand, if the number of edges between groups is significantly less than we expect by chance, or equivalently if the number within groups is significantly more, then it is reasonable to conclude that something interesting is going on [46].”

Among the many methods used to detect communities in graphs, the modularity optimization algorithm of Newman is one of the most efficient and accurate to date [46]. This method uses modularity, a quantitative measure of the observed versus expected intra-community connections, as a means to guide assignments of nodes into communities. We applied the modularity optimization algorithm to the group connectivity matrices derived from the sliding boxcars described above.

**Figure 3:**
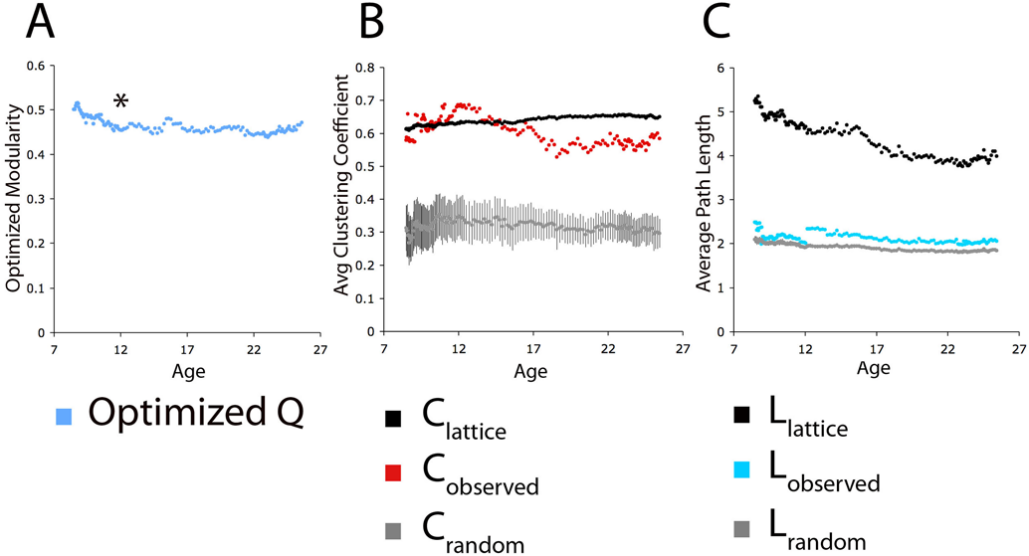
Modularity and ‘small world’ topology remain constant over age. In this figure a modularity optimization algorithm is applied, and average clustering coefficients and average path lengths are calculated for each average matrix of the ‘sliding boxcar’ across age (see Materials and Methods). A threshold of r≥0.1 was applied to the matrices before calculations were performed and denotes connected versus non-connected region pairs (see Materials and Methods). (A) From childhood through adulthood modularity (Q) remains high and constant. This result is not dependent on a specific threshold as shown in Figure S1. (Note: All age graphs to the right the asterisk show 100% graph connectedness, meaning there is a path between every node in the network. Graphs to the left of the asterisk are 78% graph connected, on average. For details see Materials and Methods and Figure S1). (B) Relative to equivalent lattice and random networks, average clustering coefficients remain high across age and do not appear to be different between children and adults. (C) Relative to equivalent lattice and random networks, average path lengths remain low across age and do not appear to be different between children and adults. High clustering coefficients and short path lengths suggest a ‘small world’ organization that does not change across the age range studied here. 95% confidence intervals are also plotted for clustering coefficients and path lengths for the generated random graphs.

**Figure 4:**
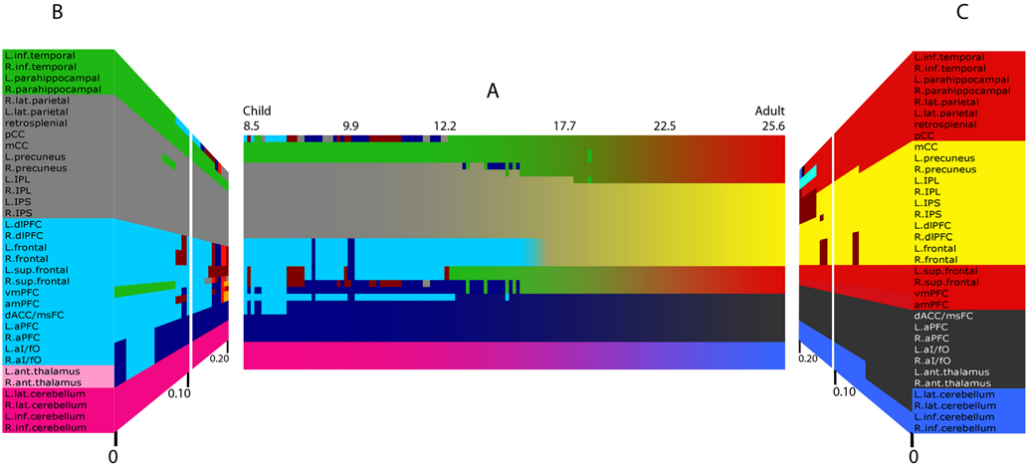
Despite high modularity in both children and adults, community assignments change over age. As in Figure 3, a modularity algorithm was applied to each matrix of the sliding boxcar across age (A) and with varying thresholds (B, C). Region legends are color coded by anatomy on the left and by adult functional network on the right (colors match Figure 2). (A) The left side of the box represents the community assignments for the youngest subjects (i.e., subjects 1–60), and the right side of the box represents the community assignments for the oldest subjects (i.e., subjects 151–210) - an age scale is presented at the top. As can be seen in the left of panel A, the modularity algorithm divided regions into communities arranged by anatomical proximity. Over age this organization transitions into modules arranged by adult functional properties. For this central panel a threshold of r≥0.1 was used to denote connected versus non-connected region pairs. (B) Community assignments of the youngest boxcar (subjects 1–60), at thresholds ranging from 0 to 0.20. Regardless of threshold regions are largely organized by anatomical proximity in this youngest age group. (C) Community assignments of the oldest boxcar (subjects 151–210), at thresholds ranging from 0 to 0.20. Regardless of threshold regions are largely organized by adult function in this oldest group.

Measures of modularity (Q) were high, and did not show large changes across the age range (Figure 3A and Figure S1 and Figure S2). This result was not dependent on any particular threshold (Figure S1). Although comparable community structure was detected at all ages examined, the components of the communities varied by age. As per our qualitative approach described above, in children, region clusters were largely arranged by cerebral lobe; while in adults, regions were largely clustered by their adult functional properties (Figure 4A). Again, this result was not unique to any particular threshold (Figure 4B and 4C) or size of boxcar (Figure S3). We do note, however, that limited data points (i.e., subjects) are available between the ages of 16 and 19 years (see Materials and Methods) and that our estimate of the specific transitions within this period should be interpreted with care.

### Over development, functional connections seem to evolve progressively along a “local to distributed” organizational axis

**Figure 5:**
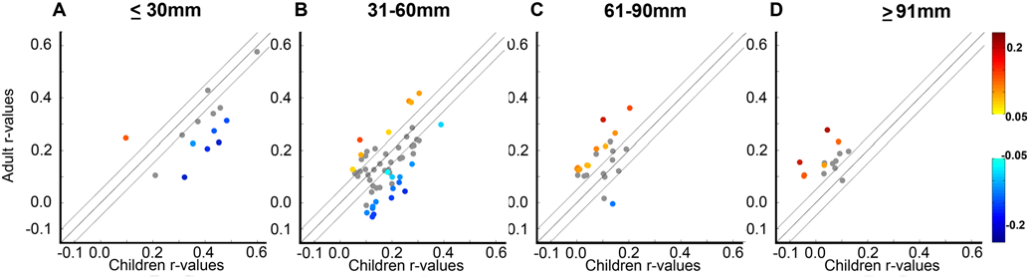
The “local to distributed” maturation is supported by a general decrease in functional connections between regions close in space, an increase in functional connection between regions distant in space, and the maintenance of several short and long-range connections that do not change with age. In this figure, functional connections are divided based on distance. Short-range functional connections are in (A,B), long-range functional connections (C,D) (y-axis, adult r-values; x-axis child r-values). Warm colors represent functional connections that are significantly greater in adults than children. Cool colors represent functional connections that are significantly greater in children than adults. Functional connections that do not significantly change with age are plotted in grey. As can be seen in (A,B), the majority of short-range functional connections that significantly change with age tend to decrease. The majority of long-range functional connections (C,D) that significantly change with age increase over time. However, many long and short-range functional connections do not significantly change over age (grey). In addition, while few, some long and short-range functional connections go against the general trend of short-range connections “growing down” and long-range functional connections “growing up.” See Figure S2 for an extended version of this figure, which includes a visualization of these functional connections on a semi-transparent brain.

As previously reported [22],[34], the segregation of closely apposed regions and the integration of distributed functional networks is associated with a general decrease in correlation strength between regions close in space and an increase in correlation strength between many regions distant in space. This trend is shown in Figure 5 and also Figure S4. Long-range functional connections tend to be weak, but increase over time (warm colors above the diagonal in Figure 5C and 5D and Figure S4C and S4D), integrating distant regions into functional networks. Short-range functional connections tend to be stronger (i.e., higher correlation strength) in children, yet those regions that do change predominantly become weaker over age (cool colors below the diagonal in Figure 5A and 5B and Figure S4A and S4B).

However, there are some interesting nuances to this trend that deserve mention. For instance, not all short-range functional connections decrease in strength over age (Figure 5A and 5B and Figure S4A and S4B). While few, some of the short-range functional connections, typically those in the same network, increase in strength over age (Figure 5A and Figure S4A). Similarly, although many long-range functional connections increase in strength, many others do not statistically change across development (Figure 5C and,5D and Figure S4C and S4D, grey connections).

### ‘Small world’ network properties are present in both children and adults

In a seminal 1998 paper, Watts and Strogatz noted that the topology of many complex systems can be described as “small world”, a type of graph architecture that efficiently permits both local and distributed processing. Graphs with a regular, lattice-like structure have abundant short-range connections, but no long-range connections. Local interactions are thus efficient, but distributed processes involving distant nodes require the traversal of many intermediate connections. Conversely, completely randomly connected graphs are fairly efficient at transferring distant or long-range signals across a network, but they are poor at local, short-range information transfer.

Watts and Strogatz, and others, often describe “small world” properties with two metrics: the average clustering coefficient and average path length of a graph. The clustering coefficient measures how well connected the neighbors of a node are to one another. The average path length measures the average minimum number of steps needed to go between any two nodes. Lattices, optimized for local processes, have high average clustering coefficients but long average path lengths. Conversely, random graphs, which have no preference for short-range connections, have low average clustering coefficients *and* short average path lengths, making them well suited for communication between distant nodes. One of Watts & Strogatz's key insights was that by randomly rewiring a relatively small number of connections in a lattice graph (i.e., introducing a few long-range connections), a graph could retain its high average clustering coefficient, but dramatically reduce its average path length, thereby enabling efficient short- *and* long-range processes. It is this hybrid graph topology (i.e., high clustering coefficients and short path lengths) that matches the observed “small world” networks in many complex systems [47].

As previously reported [21],[48],[49], relative to comparable lattice and completely random graphs, the adult graph architecture showed high clustering coefficients and short path lengths, consistent with the ‘small world’ architecture (Figure 3B and 3C). Interestingly for these networks, in children (i.e., as early as age 8), these metrics were quite similar to adults (Figure 3B and 3C), and over age there was very little change in path lengths and clustering coefficients relative to comparable random and lattice graphs. It was originally anticipated that path lengths would decrease over age as long-range anatomical connections were added. Yet even at the youngest ages examined, path length was already quite short, near those of random graphs. Importantly, these results were not dependent on any particular threshold (Figure S5). We note that while the results shown here are largely descriptive, the error bars provided in Figure 3B and 3C constructed from random graphs underscores the difference between random configurations and the observed trends.

## Discussion

The combination of graph theoretic analyses and rs-fcMRI allowed for the examination of the dynamic relationships between multiple networks over development. In the current manuscript, we examined four networks - the cingulo-opercular, fronto-parietal, cerebellar, and default mode networks. As illustrated by qualitative observations in Figure 2 (and Video S1) and modularity analysis in Figure 4, locally organized groups of regions “segregate” over development into multiple distributed adult functional networks, while the functional networks themselves “integrate.” These results support the hypothesis that functional brain development proceeds from a “local” to “distributed” organization. However, despite the “local to distributed” developmental trend, ‘small world’ organizational properties are present in both 7–9 year old child and adult graph architecture.

In the following section, these results are discussed considering two postulates: (1) the temporal pattern of spontaneous activity measured by rs-fcMRI represents a history of repeated co-activation between regions, and (2) the brain attempts to use the most efficient processing pathways available when faced with specific processing demands.

### rs-fcMRI may reflect an interaction between the maturing neural substrate and the use of efficient pathways for general task completion

As early as 1875 spontaneous synchronized neural activity has been used to study various aspects of adult brain organization [50]–[53]. However, despite the passing of over 130 years since its initial use, there remains uncertainty as to the role of intrinsic spontaneous brain activity in brain function. In adults, spontaneous correlated activity has been suggested to be important for gating information flow [54], building internal representations [43],[44],[54], and maintaining mature network relationships [43],[44],[54]. Much less work has been done in regards to development, but there are suggestions that spontaneous activity is important for the establishment of early cortical patterns (e.g., ocular dominance columns) [55]–[58] and may over time represent (in a Hebbian sense) a history of repeated co-activation between regions [21],[22],[27],[32],[34],[59],[60]. Within this framework, the changes in the correlation structure of spontaneous activity over development seen in this report may provide insight regarding the arrangement by which brain regions are communicating in children compared to adults.

If we consider the previously mentioned postulates, our results suggest that, typically, the most efficient way for children to respond to processing demands is to utilize more “local” level interactions as compared to adulthood. That is, in childhood there is, relatively greater co-activation of anatomically proximal regions than for adults with similar processing demands. A clear example of this is seen in Brown et al. [3], where identical task performance on lexical processing tests strongly activates a large set of visual regions in children, but strong visual activation is much more restricted in adults. These relationships may be reflected in correlated spontaneous activity measured via rs-fcMRI. The correlations in our youngest children would then represent the anatomical and spontaneous activity-defined initial regional relationships plus 7 years of experience-dependent Hebbian processes tuning these developing connections.

#### Changes in the neural substrate occur concurrently with changes in resting state functional connectivity

If the correlations we find in children already represent 7 years of experience-driven tuning, why should additional experience lead to a distributed solution? Under the current proposal, it is not clear then why resting state functional connectivity would change so dramatically over the reported age range. One could argue that the general experiential environment and processing demands systematically change to encourage increasing use of long-range, distributed processing relationships. We believe, however, that at least part of the explanation lies in the interaction of these “environmental demands” with maturational changes of the neural substrate.

By approximately 9 months of age the elaboration of most, if not all, short and long-range axonal connections between brain regions is thought to be complete [61]. However, synapse formation, the tuning of synaptic weights, synaptic pruning, and myelination all have unique developmental timecourses that extend further into development. For instance, from approximately 30 weeks gestation through the first two postnatal years there is substantial growth in the number of synaptic contacts throughout the cortex [62]. This growth is followed by a protracted period of synaptic pruning that reaches adult levels in the late second decade of life [63]–[65]. Importantly, pruning is selective, not random. Pruning is also largely activity dependent, and is considered critical in the differentiation of distinct functional areas [56],[66],[67].

Another commonly referenced postnatal event is myelination. As with synaptic pruning, myelination continues to occur through young adulthood. Increased myelination is thought to proceed from primary sensory and motor regions to association areas [68]–[71], roughly following the hierarchical organization introduced by Felleman and Van Essen [72] (Note that while the most frequently referenced neuroanatomical changes that occur throughout development have been highlighted here, there are several others that deserve consideration [62], [73]–[75]).

#### Changes in the neural substrate over development may lead to more efficient neural pathways for general task completion

Considering the continually changing nature of the neural substrate over development, a context for changes in rs-fcMRI can be created. For instance, as previously mentioned, increased signal propagation through the addition of a myelin sheath likely allows for more efficient communication between distant regions [22],[32],[34],[76]. Such facilitated communication may promote interactions between brain regions that, previously, had substantially less efficient communication, allowing for a more effective “solution” to any particular set of processing demands. In addition, as new, more efficient, pathways become prominent, older inefficient connections likely decrease in use, leading to experience/activity-dependent decreases of specific area-area connection strengths.

In other words, as myelination continues through development and allows for more effective long-distance neural pathways, repeated co-activation becomes more prevalent between many distant regions, and less so between many locally aligned regions, thus changing synaptic efficiencies. The statistical histories of such interactions, stored as relative synaptic weights, are then revealed via rs-fcMRI, and would lead to the “local to distributed” organization principle seen here.

It is important to note, however, that improved communication between distant regions (via myelination) would not necessarily cause a wholesale decrease in connections that were originally organized locally. Many of these local connections likely continue to contribute to the most efficient “solution” for any particular task and remain in use. In fact, the change in dynamics may actually contribute to distinct local connections increasing with time. This possibility may underlie the increases in strength of specific short-range connections seen in Figure 5 and Figure S2.

Along the same lines, as Fuster [77] has pointed out, we note that myelination is not an indispensable property of utilized axons. Unmyelinated axonal connections are still quite capable of transmitting information. For this reason, the first 7 years of experience dependent statistical learning may indeed result in increases in long distance functional connections well before mature myelination is in place, an idea consistent with the short average path lengths found in even the youngest networks we examined (Figure 3). Thus, it is not surprising that some long-distant functional connections are present in children and do not statistically change with age (Figure 5 and Figure S2).

We note that recent results in the aging literature suggest that many of the trajectories observed in the current manuscript continue inversely with advancing age [24],[78]. That is, with aging, the functional organization, revealed via rs-fcMRI, becomes less distributed and more local. Thus the dynamic interactions we describe here likely continue as part of normal senescence [78].

### The results presented here are consistent with other views of functional brain development

The “local to distributed” organizing principle resonates with recent suggestions that perceptual and cognitive development involve the simultaneous segregation and integration of information processing streams [1],[22],[76],[79],[80]. For instance, the “interactive specialization” hypothesis advanced by Johnson and colleagues, is consistent with these findings [1], [81]–[83]. Johnson points out that cortical regions and pathways have biased information processing properties at birth due to anatomic connectivity, yet they are much less selective than in adults (i.e., they are “broadly tuned”).

Interactive specialization predicts that shortly after birth, large sets of regions and pathways will be partially active during specific task conditions, However, as these pathways interact and compete with each other throughout development, selected regions will come online, be maintained, or become selectively activated or “tuned” as particular pathways dominate for specific task demands. Thus, regional specialization relies on the evolving and continuous interactions with other brain regions over development. If one extends this framework to the network level, the increases, decreases, and maintenance of correlation strengths seen between regions may reflect “specialization” of specific neural pathways to form the functional networks seen in adults.

### Graph analysis suggests that small world properties are present in late childhood

The “local to distributed” developmental trajectory, discussed above, seems to be driven by an abundance of local, short range connections that generally decrease in strength over age as well as distant, long range connections that generally increase in strength over age. Given the more prevalent short-range connections in children, we expected a more lattice-like structure, with high clustering coefficients and relatively high path lengths. The results, however, clearly indicated that path lengths were near those of equivalent random graphs, and that the child functional networks are already organized as small world networks.

This result can be explained in the context of the re-wiring procedure discussed by Watts and Strogatz [47]. Randomly rewiring a small percentage of local connections in a lattice has a mild linear effect on clustering coefficients, but a highly non-linear effect on path lengths. This is to say, that by rewiring a small fraction of a lattice's connections, substantial drops in path lengths can be seen, with almost no change in the clustering coefficient. In late childhood, as shown in Figure 5 and Figure S2, there are already a significant number of long-range short cuts present. These long-range functional connections are likely responsible for the relatively short path lengths in the child group. We anticipate that if the developmental trajectory of short and long-range functional connections were extended to younger ages, fewer long-range ‘short-cut’ functional connections would be present, and more short-range functional connections would exist. Hence, the path lengths at these younger ages (<7 years old) would likely be longer. Nevertheless, by 8 years old, the networks already display ‘small world’ properties similar to those of adult networks, indicating that efficient graph structures are already in place for both local and distant processing, though they are organized differently than in later development.

While we identified small world properties in both child and adult graphs, the size of the graph is relatively small with only 34 nodes. Therefore, it is possible that with an increased number of nodes the specific results identified here will change, a possibility that will be addressed in further studies.

### Need for generalization to other regions and modalities

The regions used in the present analyses were all derived from adult imaging studies. It seems likely that additional regions may be included in one or more of these networks in childhood. In addition, individual differences with regards to the regions and networks chosen likely exist. Future work that includes regions derived from studies using a child population and obtaining the functional connections within subjects from individually defined functional areas may refine the networks and developmental timecourses presented here [84].

Of note, resting-state functional connectivity has been reported to be constrained by anatomical distance (i.e., correlations between regions decrease as a function of distance following an inverse square law) [85]. Thus, if a shift in this general bias occurred with development, then it is feasible that some of the changes seen here could be related to such a shift. With this said, the specificity of the connection changes observed over age, the number of connections that run opposite to the general trends, and the similarity of the distance relationship in connectivity between children and adults when plotting all possible connections (see Figure S6), all suggest that the majority of changes observed here are not related to changes in this bias. In addition, while there are now reports suggesting that changes observed over development with blood oxygen level dependent (BOLD) fMRI are not the product of changes in hemodynamic response mechanisms over age [86],[87], differences in the hemodynamic response function between children and adults could conceivably affect our results [88].

A limitation of rs-fcMRI in general is the restricted frequency distribution that can be examined. rs-fcMRI is used to measure correlations in a very low frequency range, typically below 0.1 Hz. Dynamic changes in correlations in other frequency distributions could exist (for example see [89]). It is also possible that there are undetected developmental changes in power across frequency bands orthogonal to the changes visualized here. The combination of other imaging and psychometric techniques with rs-fcMRI will likely help address these considerations. Characterizing additional networks and how these changes map onto behavior will also help further characterize functional brain development. Specifically, future work that demonstrates a direct relationship between behavior and the developmental trajectory seen here with rs-fcMRI, is presently needed to confirm (or reject) many of the theories presented here and elsewhere. Importantly, consideration of these issues need not be limited to developmental studies, but should be considered whenever investigators compare groups with rs-fcMRI.

Nonetheless, the general results presented here represent a strong set of hypotheses to be tested in broader domains and larger-scale brain graphs. First, that by age 8 years, regional relationships, as defined by rs-fcMRI, are organized as small-world-like networks, which, relative to adults, emphasize local connections. Second, that for the same regions, adult networks show similar network metrics but with regional relationships that have a longer-range, more distributed structure reflecting adult functional histories. In other words, the modular structure of large-scale brain networks will change with age, but even school age children will show relatively efficient processing architecture.

## Materials and Methods

### Subjects

Subjects were recruited from Washington University and the local community. Participants were screened with a questionnaire to ensure that they had no history of neurological/psychiatric diagnoses or drug abuse. Informed consent was obtained from all subjects in accordance with the guidelines and approval of the Washington University Human Studies Committee.

### Data acquisition and pre-processing

fMRI data were acquired on a Siemens 1.5 Tesla MAGNETOM Vision system (Erlangen, Germany). Structural images were obtained using a sagittal magnetization-prepared rapid gradient echo (MP-RAGE) three-dimensional T1-weighted sequence (TE = 4 ms, TR = 9.7 ms, TI = 300 ms, flip angle = 12 deg, 128 slices with 1.25×1×1 mm voxels). Functional images were obtained using an asymmetric spin echo echo-planar sequence sensitive to blood oxygen level-dependent (BOLD) contrast (volume TR = 2.5 sec, T2* evolution time = 50 ms, α = 90°, in-plane resolution 3.75×3.75 mm). Whole brain coverage was obtained with 16 contiguous interleaved 8 mm axial slices acquired parallel to the plane transecting the anterior and posterior commissure (AC-PC plane). Steady state magnetization was assumed after 4 frames (∼10 s).

Functional images were first processed to reduce artifacts [23],[90]. These steps included: (i) removal of a central spike caused by MR signal offset, (ii) correction of odd vs. even slice intensity differences attributable to interleaved acquisition without gaps, (iii) correction for head movement within and across runs and (iv) within run intensity normalization to a whole brain mode value of 1000. Atlas transformation of the functional data was computed for each individual via the MP-RAGE scan. Each run then was resampled in atlas space (Talairach and Tournoux, 1988) on an isotropic 3 mm grid combining movement correction and atlas transformation in one interpolation [91],[92]. All subsequent operations were performed on the atlas-transformed volumetric timeseries.

### rs-fcMRI pre-processing

For rs-fcMRI analyses as previously described [16],[23], several additional preprocessing steps were used to reduce spurious variance unlikely to reflect neuronal activity (e.g., heart rate and respiration). These steps included: (1) a temporal band-pass filter (0.009 Hz<f<0.08 Hz) and spatial smoothing (6 mm full width at half maximum), (2) regression of six parameters obtained by rigid body head motion correction, (3) regression of the whole brain signal averaged over the whole brain, (4) regression of ventricular signal averaged from ventricular regions of interest (ROIs), and (5) regression of white matter signal averaged from white matter ROIs. Regression of first order derivative terms for the whole brain, ventricular, and white matter signals were also included in the correlation preprocessing. These pre-processing steps likely decrease or remove developmental changes in correlations driven by changes in respiration and heart rate over age.

### Extraction of resting state timeseries

Resting state (fixation) data from 210 subjects (66 aged 7–9; 53 aged 10–15; 91 aged 19–31) were included in the analyses. For each subject at least 555 seconds (9.25 minutes) of resting state BOLD data were collected. 34 previously published regions comprising 4 functional networks (i.e., cingulo-opercular, fronto-parietal, cerebellar, and default networks; see Table 1 and Figure 1) were used in this analysis [16],[21],[22],[37]. For each region, a resting state timeseries was extracted separately for each individual. For 10 adult subjects, resting data was continuous. For the remaining 200 subjects, resting periods were extracted from between task periods in blocked or mixed blocked/event-related design studies [22]. These concatenated-extracted rest periods were shown to be equivalent to continuous resting data in a recent study describing this method [23]. In addition, several previous findings using this technique [21],[22],[32] have now been replicated using continuous resting blocks [27],[33],[34] and other continuous resting data [89].

### Generation of average group correlation matrices across development

To examine the functional connections within and between the large set of regions used in this manuscript we chose to use graph theory. Graph theory is particularly well suited to study large-scale systems organization across development, but requires the data be organized into specific correlation matrices. To do this, for each of the 210 subjects, the resting state BOLD timeseries from each region was correlated with the timeseries from every other region, creating 210 square correlation matrices (34×34). Average group matrices were then created using a sliding boxcar grouping of subjects in age-order (i.e., group1: subjects 1–60, group2: subjects 2–61, group3: subjects 3–62, … group151: subjects 151–210), thus generating a series of groups with average ages ranging from 8.48 years old to 25.48 years old with each group composed of 60 subjects. Average correlation coefficients (r) for each group were generated from the subjects' individual matrices using the Schmidt-Hunter method for meta-analyses of r-values [21],[85],[93]. In cases when the terms “child” or “adult” are used, the matrices or results referred to are the first and last of the sliding boxcar groups respectively, i.e., the child group is the youngest 60 subjects, with an average age of 8.48 years old, and the adult group is the oldest 60 subjects, with an average age of 25.48 years old.

### Spring-embedded graph theoretic layout and visualization

To generate a dynamic representation of the functional connections between regions across development, each of the groups' correlation matrices was converted into a thresholded graph, such that correlations higher than r≥0.1 were considered connections, while correlations lower than the threshold were not connections.

For our initial analyses [21],[22],[32] graphs in child and adult groups were presented in either a pseudo-anatomical fashion or in their actual 3D positions (in Talairach space). Here we add another representation often used in graph theory - spring embedding. In this procedure, a spring constant is added to all of the connections in the network allowing for the pairwise regional connections to relax to their lowest energetic state. The algorithm applied in the present analysis is known as Kamada-Kawai [45] - one of the most commonly used strategies for displaying graph network data. In brief, each functional connection between a pair of nodes is treated as a spring with a spring constant related to the strength of the specific correlation. The nodes are then randomly placed in a plane, which places high strain on the “spring-loaded” connections. The algorithm then iteratively adjusts the positions of each node to reduce the total energy of the system to a minimum. As the pair-wise connections relax to their lowest energetic states the “natural” configuration of the network is revealed. By observing multiple “spring embedded” graphs across the subjects in age-order, approximately representing a 6 month temporal sliding box car (i.e., group1: subjects 1–60, group2: subjects 2–61, etc.), a movie representation can be made that shows the development of the full system (see Video S1). The interpolations, algorithm application, and movie production were performed using MATLAB (The Mathworks, Natick, MA) and SoNIA (Social Network Image Animator) [94].

### Modularity analysis

Communities for our graph were detected with the modularity optimization method of Newman [46]. The modularity, or Q, of a graph is a quantitative measure of the number of edges found within communities versus the number predicted in a random graph with equivalent degree distribution. A positive Q indicates that the number of intra-community edges exceeds those predicted statistically. A wide range of Q may be found for a graph, depending on how nodes are assigned to communities. The set of node assignments that returns the highest Q is the optimal community structure sought by the modularity optimization algorithm, which follows a recursive two-step process. First, a modularity matrix similar to a Laplacian is constructed from the nodes in question, comparing observed versus expected edges. If this matrix has a positive eigenvalue, the eigenvector of the largest eigenvalue is used to split the nodes into two subgraphs, and Q is calculated. Second, nodes are swapped individually between the two subgraphs to see if an increase in Q can be found. Once a maximal Q is found from these swaps, the process is repeated on the subgraphs. At any point in this process, if the matrix has no positive eigenvalues, or if a proposed split does not increase Q, the subgraph is set aside, and defines a community. To detect communities in our networks over a range of ages, we used the sliding boxcar group average correlation matrices described above in “Generation of average group correlation matrices across development.” With weights retained, the modularity optimization algorithm was applied to each matrix along the sliding boxcar. A range of thresholds was explored to define connections for these calculations (see Figure 4 and Figure S1). Any particular threshold did not change the conclusions presented in the main manuscript. A threshold of 0.10 was chosen to display in the main manuscript because it balances two principles: (1) eliminating a multitude of weak correlations, which may obscure more physiologically relevant correlations, and (2) retaining high graph connectedness, so that communities arise from partitioning and not thresholding. Graph connectedness captures the extent of nodes fragmented from the main graph due to increasing thresholds. It is defined for a graph of N nodes as the mean of an NxN matrix, where cell i,j is 1 if a path exists between node i and node j (self-connections are allowed), and is 0 otherwise. A graph in which all nodes can reach each other has 100% graph connectedness, whereas a fragmented network in which some nodes cannot reach the rest has a lower connectedness. The modularity optimization analysis returned a set of community assignments for the nodes, as well as the Q of the graph with those assignments. The group assignments for the nodes were converted to colors and are displayed in Figure 4. The robustness of the community assignments was also tested using a different information theoretic procedure implemented by Meila, [95], which utilizes the measure ‘variation of information (VOI)’ (see Figure S7 and also [96]). All calculations were performed in MATLAB (The Mathworks, Inc., Natick, MA).

### Characterization of connection length versus the change in correlation strength over development

To characterize the relationship between connection length and the change in correlation strength over development, we split all 561 possible connections into 4 groups based on vector distance. Since using vector distance as an approximate for connectional distance is much more inconsistent when comparing ROIs across the midline, only intrahemispheric connections or connections to midline structures (i.e., within 5 mm of the midline) were examined. These connections were then sorted by connection length and plotted on a graph where the x-axis corresponds to the child correlation strengths and the y-axis corresponds to the adult correlation strengths (Figure 5 and Figure S2). On both the graphs (Figure 5) and the cortical surfaces (Figure S2), the color of the lines denotes the strength of correlation. Significant differences seen in Figure 5 and Figure S2 were obtained via direct comparison between children (the youngest 60 children out of 210 total subjects; age 7.01–9.67; average age 8.48) and adults (the oldest 60 adults out of 210 total subjects; age 22.47–31.39; average age 25.48). Two-sample two-tailed *t*-tests (assuming unequal variance; p≤0.05) were performed on all potential connections that passed the above criteria. Fischer z transformation was applied to the correlation coefficients to improve normality for the random effects analysis. To account for multiple comparisons the Benjamini and Hochberg False Discovery Rate [97] was applied. Connections that were significantly different between groups, but r<0.1 in both groups, were not displayed.

### ‘Small world’ characterization

The small-world metrics were calculated according to descriptions by Watts and Strogatz [47]. In the main manuscript, calculations were performed on the group average correlation matrices thresholded at 0.10 and converted to binary matrices (for analysis across varying thresholds see Figure S3). For each matrix across age, the average clustering coefficient and average path lengths were compared to those values in lattices with equivalent N (number of nodes) and K (number of connections). To ensure that our matrices also differed from random graphs, 100 random graphs with equivalent degree distributions were also created. From these graphs mean average path lengths and clustering coefficients were calculated. These metrics are presented in Figure 3 and Figure S3. All calculations were performed in MATLAB (The Mathworks, Natick, MA).

## Supporting Information

Figure S1 Modularity remains relatively high across age and does not differ between children and adults across differing thresholds. Blue dots represent modularity and red dots represent graph connectedness. A graph in which there is a path between all nodes represents 100% graph connectedness, whereas a fragmented network in which some nodes cannot reach the rest has a lower graph connectedness (see Materials and Methods for details). (A) Modularity across age as presented in Figure 3 of the main manuscript. (B) Modularity across thresholds for children. (C) Modularity across thresholds for adults.
(0.50 MB TIF)

Figure S2 Scatterplot of modularity as a function of age. Each point in the graph represents the modularity calculated for each individual subject. A threshold of r≥0.1 was applied to each subject's matrices before calculations were performed and denotes connected versus non-connected region pairs (see Materials and Methods).
(0.35 MB TIF)

Figure S3 Reducing the boxcar size does not substantially alter community assignments over age. The same procedure as presented in Figure 4 with the boxcar reduced to (A) 40 subjects and (B) 20 subjects.
(4.65 MB TIF)

Figure S4 An extended version of Figure 5, which includes a visualization of these connections represented on a semi-transparent brain.
(4.69 MB TIF)

Figure S5 Clustering coefficients and path lengths do not differ between children and adults across differing thresholds with respect to comparable lattice and random graphs. For children all parameters across thresholds were calculated from the first 60 subjects in age order (i.e., subjects 1–60, average age 8.48). For adults, all parameters across thresholds were calculated from the last 60 subjects in age order (i.e., subjects 151–210, average age 25.48. (A) Clustering Coefficients across thresholds for children compared to equivalent lattice and random networks. (B) Path lengths across thresholds for children compared to equivalent lattice and random graphs. (C) Clustering Coefficients across thresholds for adults compared to equivalent lattice and random graphs. (D) Path lengths across thresholds for adults compared to equivalent lattice and random graphs. At all thresholds examined, both children and adults show relatively high clustering coefficients and low path lengths, consistent with ‘small world’ topology.
(0.59 MB TIF)

Figure S6 Connection strength as a function of distance for all possible connections is similar between children and adults. The relationship of correlation as a function of distance is described by the inverse square law, r∼1/D^2^, as reported in [85] for all possible connections in children (blue) and adults (red).
(0.71 MB TIF)

Figure S7 Variation of information (VOI) in observed and equivalent random networks subjected to perturbation alpha. VOI is a measure of how much information is not shared between two sets of community assignments and allows for the quantification of network robustness (see [95] and [96]). Values of 0 indicate identical community assignments, and values of 1 indicate maximally different community assignments. To assess the stability of community assignments, the edges of a network are randomized with probability alpha to perturb the network, and the VOI between the original and perturbed networks are calculated over a range of alpha. An equivalent random network was generated for comparison. The entire perturbation process was repeated 50 times to obtain mean VOI values and standard errors of the means, which are plotted as error bars. (A) VOI over a range of alpha in the youngest boxcar and equivalent random graphs. (B) VOI over a range of alpha in the oldest boxcar and equivalent random graphs. Compared to random graphs the community assignments in both children and adults are significantly robust.
(0.43 MB TIF)

Video S1 Over age, the graph architecture matures from a “local” organization to a “distributed” organization. This movie shows the dynamic development and interaction of positive correlations between the two task control networks, the default network, and cerebellar network using spring embedding. The figure highlights the segregation of local, anatomically clustered regions and the integration of functional networks over development. This is the full movie that Figure 3 is based on in the main text. Nodes are color coded by there adult network profile (core of the nodes) and also by there anatomical location (node outlines). Black - cingulo-opercular network; Yellow - fronto-parietal network; Red - default network; Blue - cerebellar; Light blue - frontal cortex; Grey - parietal cortex; Green - temporal cortex, Pink - cerebellum, Light pink - thalamus. At the beginning of the movie (i.e. in children) regions are largely organized by their anatomical location, but over age anatomically clustered regions segregate. The cluster of frontal regions (light blue outlines) best demonstrates this segregation. In addition, at the beginning of the movie (i.e., in children) the more distributed adult functional networks (core colors of nodes) are in many ways disconnected; however, over development the functional networks integrate. The isolated regions of the default network in childhood (Red) that coalesce into a highly correlated network best illustrate this integration. Over age node organization shifts from the “local” arrangement in children to the “distributed” organization commonly observed in adults.
(9.68 MB MP4)

Video S2 Reducing the boxcar size to 40 subjects does not change qualitative patterns observed with the 60 subject boxcar. The same procedure as presented in Figure 3 and Video S1 is presented here with the boxcar reduced to 40 subjects.
(9.62 MB MPG)
