# Supplementary figures and images for: Functional Brain Networks Develop from a “Local to Distributed” Organization
Source: PLoS Comput Biol. 2009 May 1;5(5):e1000381. doi: 10.1371/journal.pcbi.1000381 (PMC2671306; doi:10.1371/journal.pcbi.1000381)

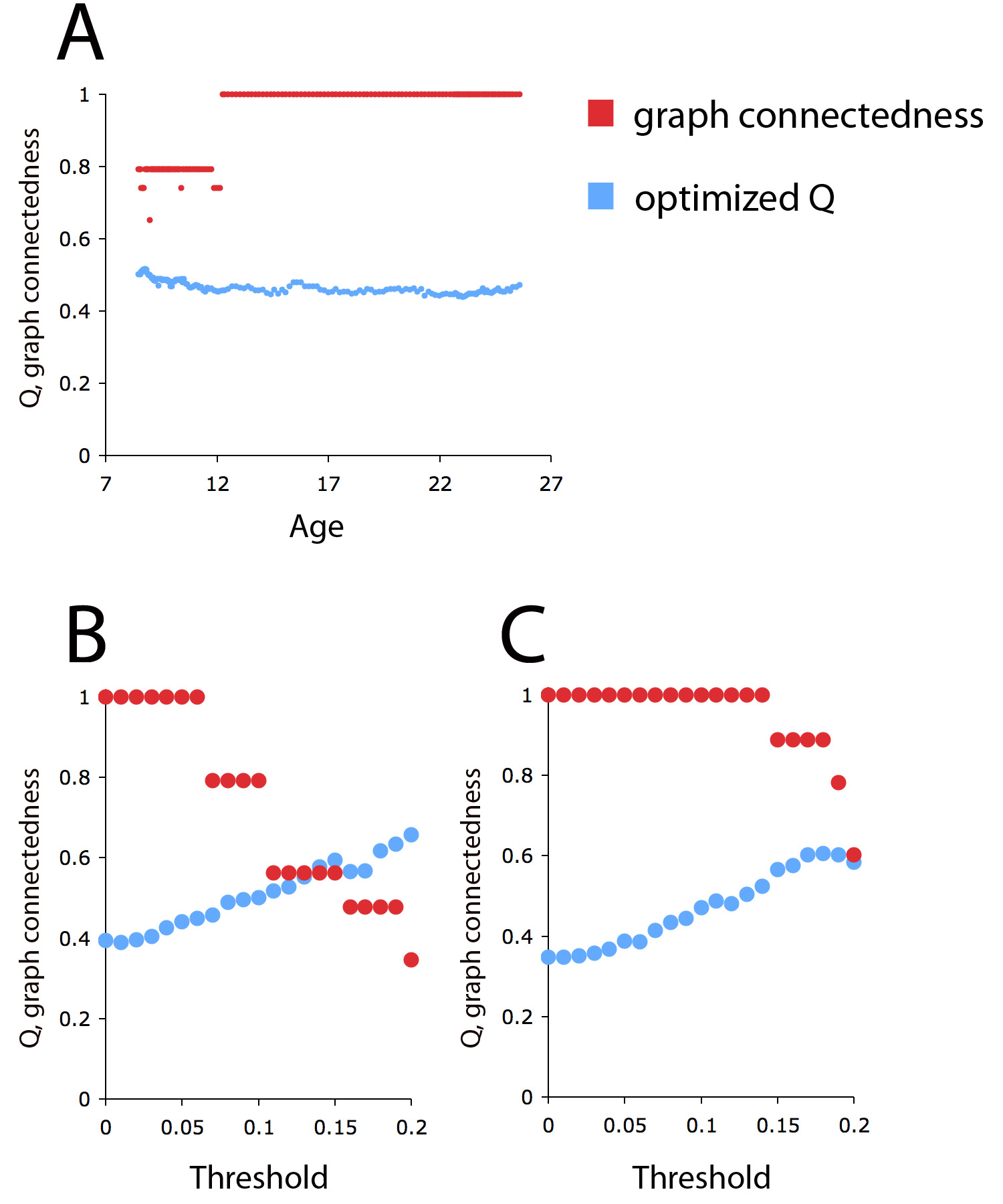

Supplement: Figure S1 — Modularity remains relatively high across age and does not differ between children and adults across differing thresholds. Blue dots represent modularity and red dots represent graph connectedness. A graph in which there is a path between all nodes represents 100% graph connectedness, whereas a fragmented network in which some nodes cannot reach the rest has a lower graph connectedness (see Materials and Methods for details). (A) Modularity across age as presented in Figure 3 of the main manuscript. (B) Modularity across thresholds for children. (C) Modularity across thresholds for adults. (0.50 MB TIF) [file pcbi.1000381.s001.tif]

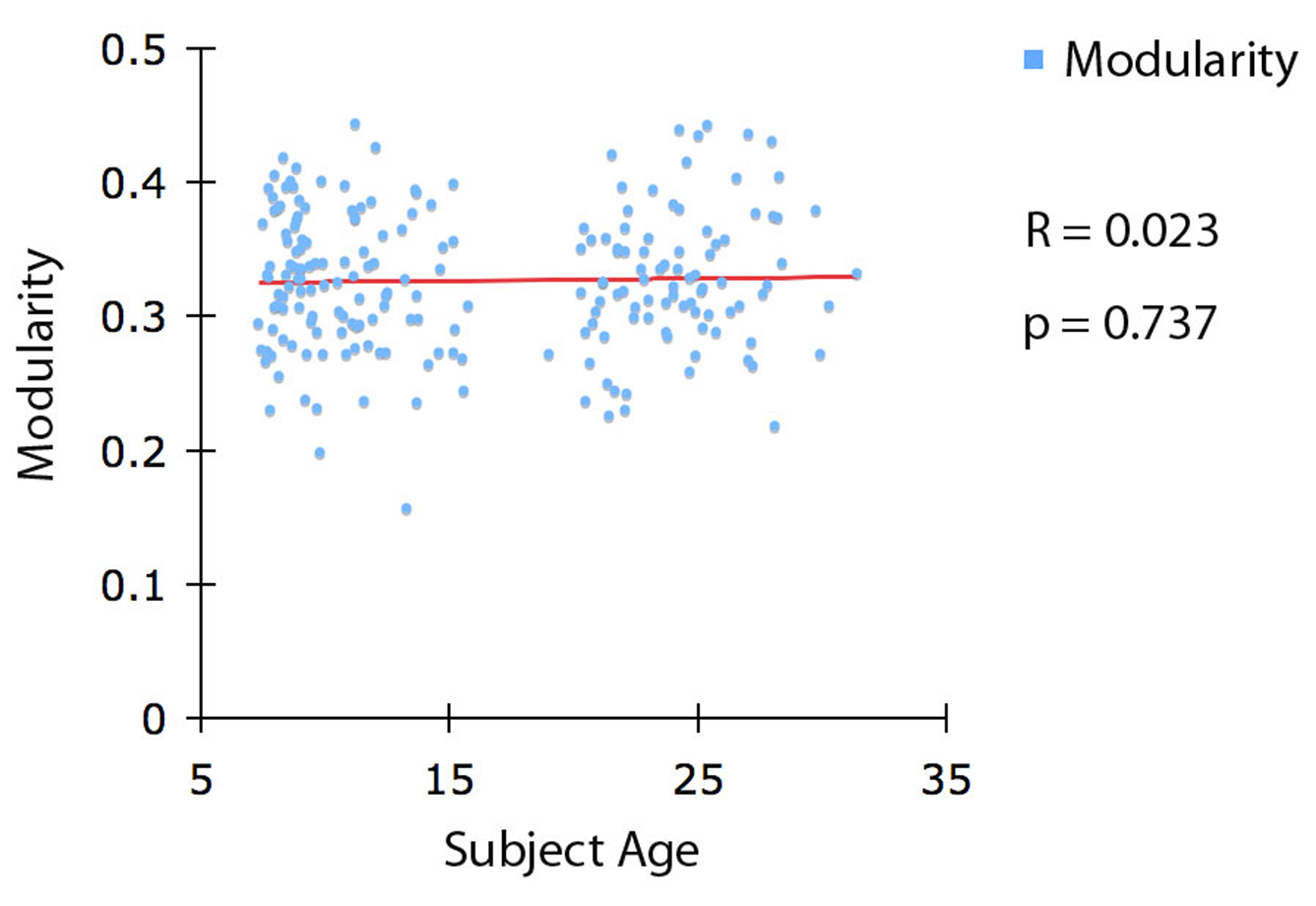

Supplement: Figure S2 — Scatterplot of modularity as a function of age. Each point in the graph represents the modularity calculated for each individual subject. A threshold of r≥0.1 was applied to each subject's matrices before calculations were performed and denotes connected versus non-connected region pairs (see Materials and Methods). (0.35 MB TIF) [file pcbi.1000381.s002.tif]

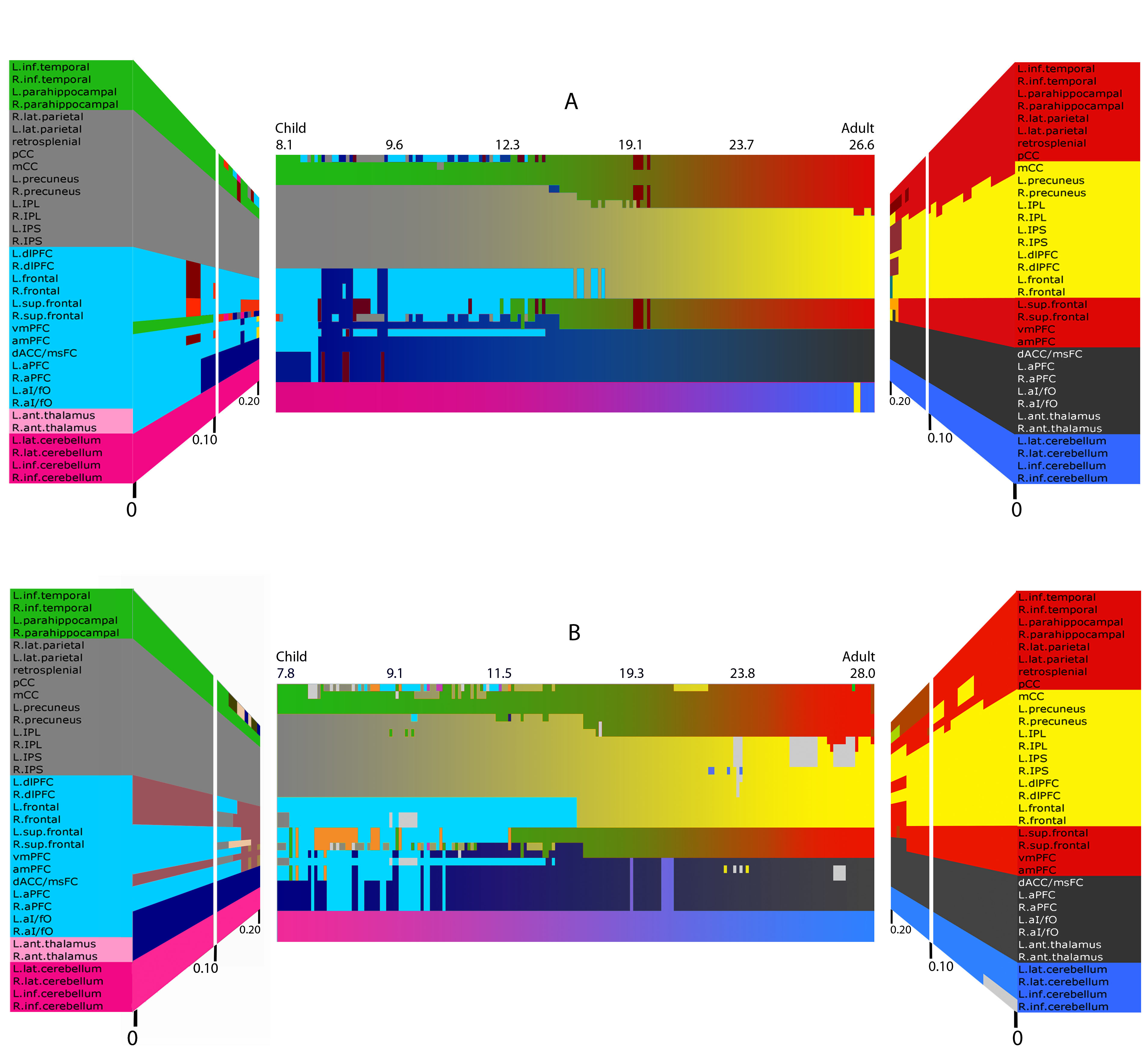

Supplement: Figure S3 — Reducing the boxcar size does not substantially alter community assignments over age. The same procedure as presented in Figure 4 with the boxcar reduced to (A) 40 subjects and (B) 20 subjects. (4.65 MB TIF) [file pcbi.1000381.s003.tif]

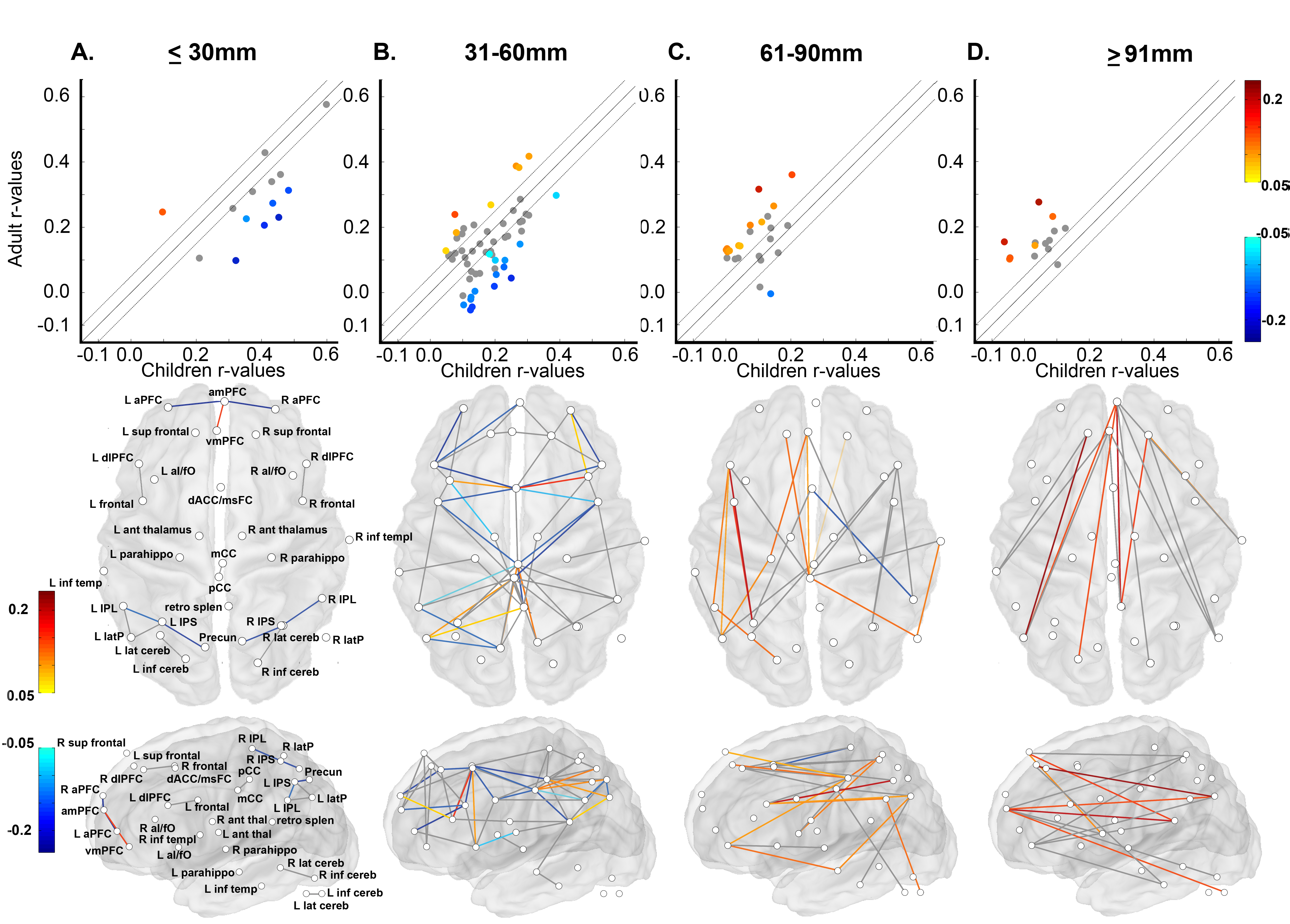

Supplement: Figure S4 — An extended version of Figure 5, which includes a visualization of these connections represented on a semi-transparent brain. (4.69 MB TIF) [file pcbi.1000381.s004.tif]

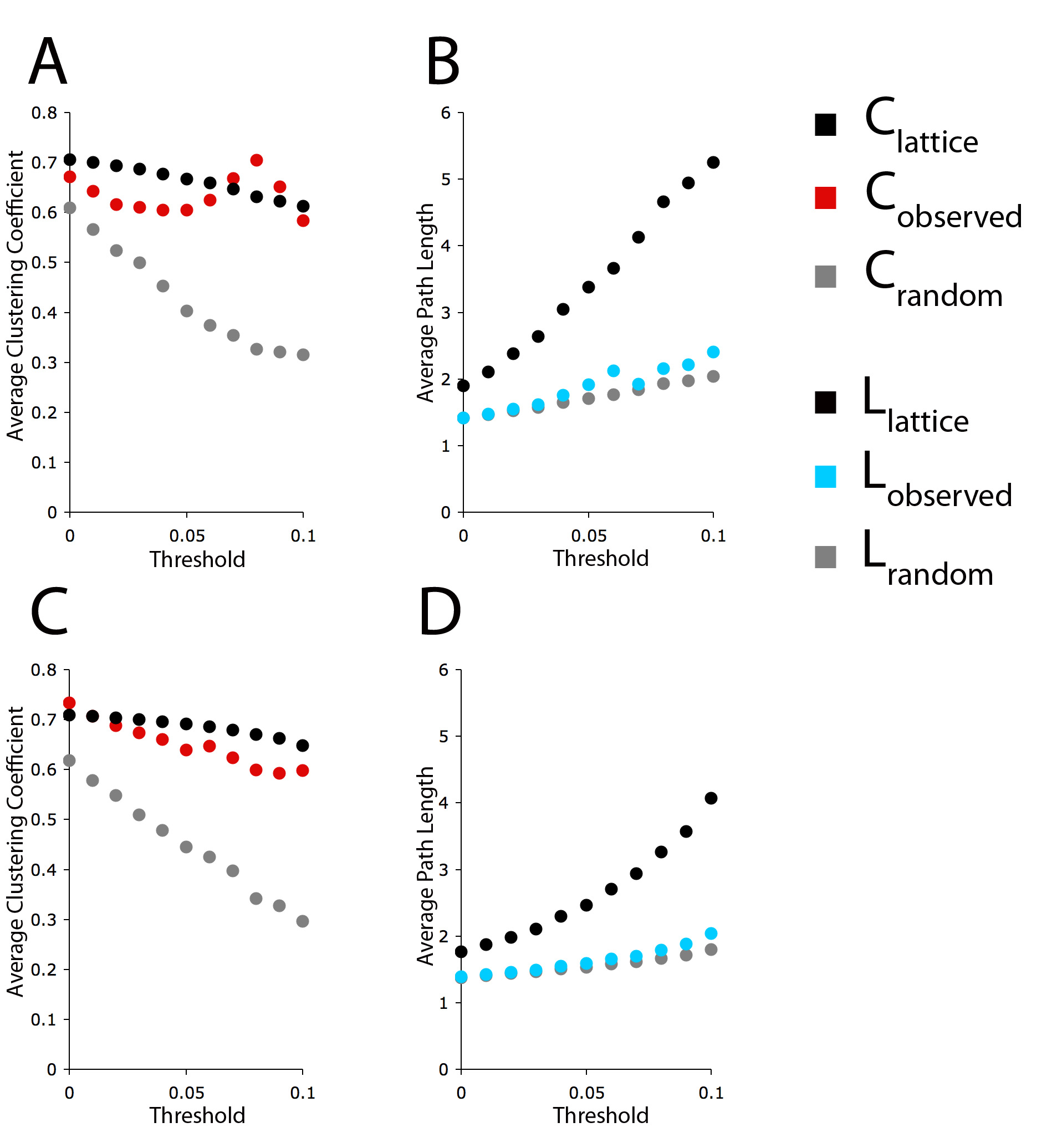

Supplement: Figure S5 — Clustering coefficients and path lengths do not differ between children and adults across differing thresholds with respect to comparable lattice and random graphs. For children all parameters across thresholds were calculated from the first 60 subjects in age order (i.e., subjects 1–60, average age 8.48). For adults, all parameters across thresholds were calculated from the last 60 subjects in age order (i.e., subjects 151–210, average age 25.48. (A) Clustering Coefficients across thresholds for children compared to equivalent lattice and random networks. (B) Path lengths across thresholds for children compared to equivalent lattice and random graphs. (C) Clustering Coefficients across thresholds for adults compared to equivalent lattice and random graphs. (D) Path lengths across thresholds for adults compared to equivalent lattice and random graphs. At all thresholds examined, both children and adults show relatively high clustering coefficients and low path lengths, consistent with ‘small world’ topology. (0.59 MB TIF) [file pcbi.1000381.s005.tif]

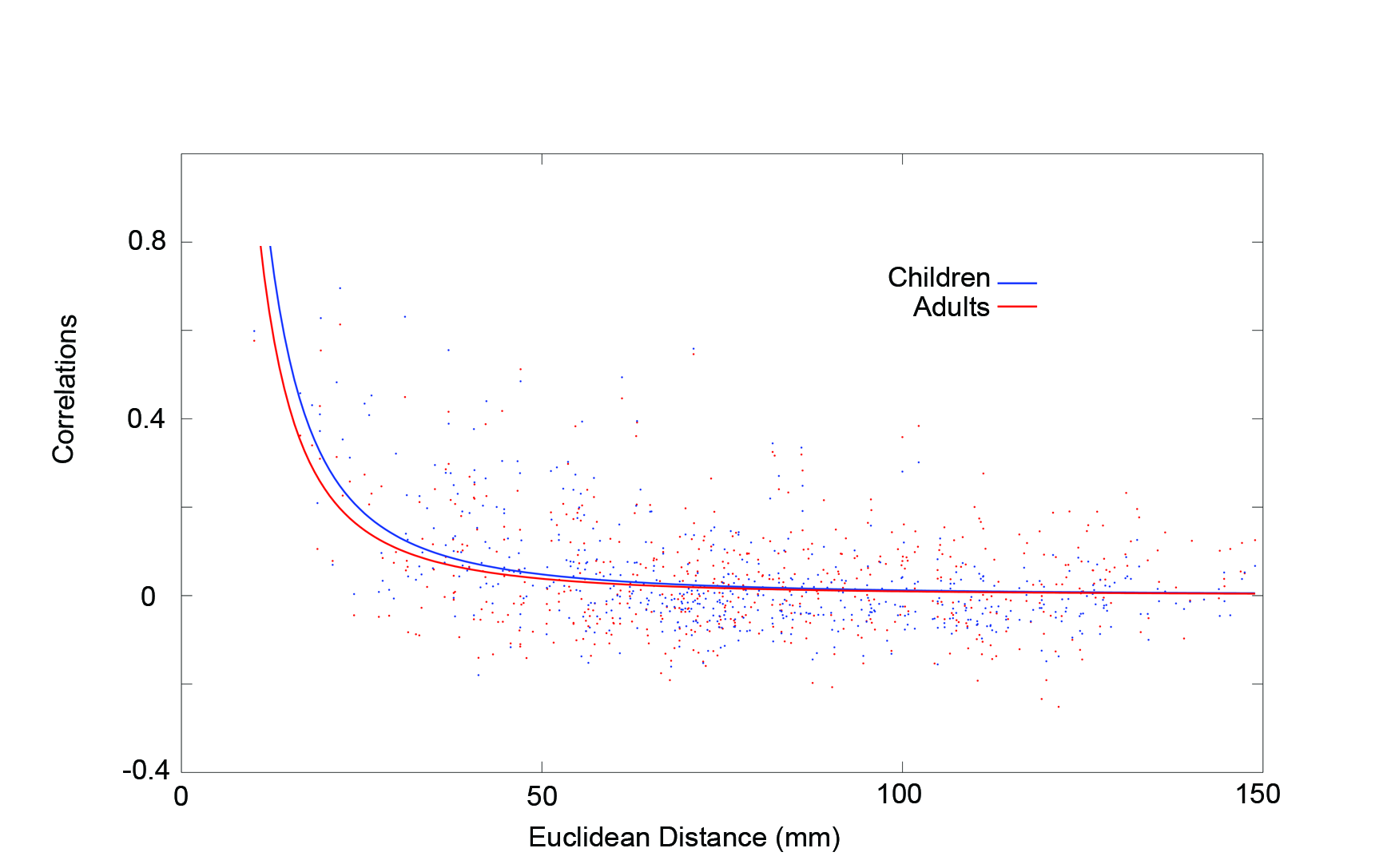

Supplement: Figure S6 — Connection strength as a function of distance for all possible connections is similar between children and adults. The relationship of correlation as a function of distance is described by the inverse square law, r∼1/D2, as reported in [85] for all possible connections in children (blue) and adults (red). (0.71 MB TIF) [file pcbi.1000381.s006.tif]

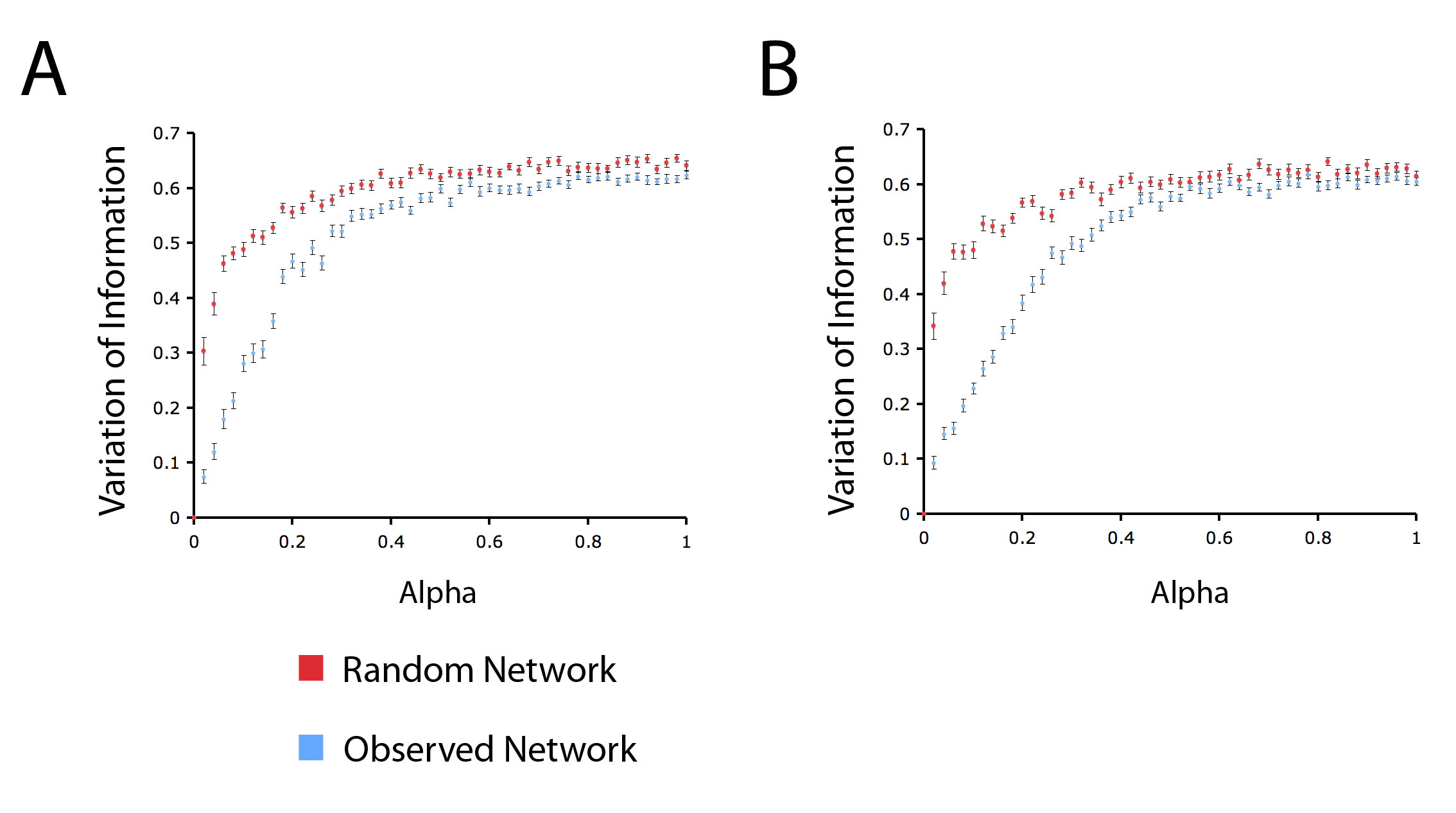

Supplement: Figure S7 — Variation of information (VOI) in observed and equivalent random networks subjected to perturbation alpha. VOI is a measure of how much information is not shared between two sets of community assignments and allows for the quantification of network robustness (see [95] and [96]). Values of 0 indicate identical community assignments, and values of 1 indicate maximally different community assignments. To assess the stability of community assignments, the edges of a network are randomized with probability alpha to perturb the network, and the VOI between the original and perturbed networks are calculated over a range of alpha. An equivalent random network was generated for comparison. The entire perturbation process was repeated 50 times to obtain mean VOI values and standard errors of the means, which are plotted as error bars. (A) VOI over a range of alpha in the youngest boxcar and equivalent random graphs. (B) VOI over a range of alpha in the oldest boxcar and equivalent random graphs. Compared to random graphs the community assignments in both children and adults are significantly robust. (0.43 MB TIF) [file pcbi.1000381.s007.tif]
